# Validation of the Target Protein of Insecticidal Dihydroagarofuran Sesquiterpene Polyesters

**DOI:** 10.3390/toxins8030079

**Published:** 2016-03-18

**Authors:** Lina Lu, Zhijun Qi, Qiuli Li, Wenjun Wu

**Affiliations:** 1 Institute of Pesticide Science, College of Plant Protection, Northwest A & F University, Yangling 712100, Shaanxi, China; linalusailboat@163.com (L.L.); qzhij@nwsuaf.edu.cn (Z.Q.); liqiuli@nwsuaf.edu.cn (Q.L.); 2 Key Laboratory of Botanical Pesticide R & D in Shaanxi Province, Yangling 712100, Shaanxi, China

**Keywords:** dihydroagarofuran sesquiterpene polyesters, target protein, target validation, V-ATPase, subunit H of V-ATPase, *Mythimna separata* Walker

## Abstract

A series of insecticidal dihydroagarofuran sesquiterpene polyesters were isolated from the root bark of Chinese bittersweet (*Celastrus angulatus* Max). A previous study indicated that these compounds affect the digestive system of insects, and aminopeptidase N3 and V-ATPase have been identified as the most putative target proteins by affinity chromatography. In this study, the correlation between the affinity of the compounds to subunit H and the insecticidal activity or inhibitory effect on the activity of V-ATPase was analyzed to validate the target protein. Results indicated that the subunit H of V-ATPase was the target protein of the insecticidal compounds. In addition, the possible mechanism of action of the compounds was discussed. The results provide new ideas for developing pesticides acting on V-ATPase of insects.

## 1. Introduction

Identification of target proteins is the basis for development of new pesticides. The discovery of novel targets may result in a series of new pesticides. Moreover, in the development of pesticides, natural products are useful probes in providing new targets. A common example is nicotine; the identification and elucidation of the molecular structure of the target protein of nicotine (*i.e.*, insect nAChR) promoted the development of neonicotinoid pesticides [1,2,3].

A series of insecticidal compounds, namely, sesquiterpene polyesters, sharing a dihydro-*b*-agarofuran sesquiterpenoid skeleton were isolated and characterized by Wu *et al.* from the root bark of *Celastrus angulatus* Max (Celastraceae) [4,5,6]. These insecticidal compounds mainly affect the digestive system of pests, presenting a series of symptoms, such as excitement, twitching, emesis, and loss of body fluid after oral administration [7,8]. Transmission electron microscopy (TEM) analysis showed that the midgut epithelial cells of *Mythimna separata* Walker larvae that ingested celangulin V (CV) were damaged, showing visible vacuolization of cytoplasm, serious disruption of microvilli, fragmentation of rough endoplasmic reticulum cisternae, and rupture of plasma membrane. Subsequently, these morphological changes induce leakage of cytoplasm contents into the midgut lumen, resulting in appearance of numerous lysosome-like vacuoles and secretion [8,9].

However, the mechanisms of action and the target protein of the dihydroagarofuran sesquiterpene polyesters remain unknown. In our previous study, 11 binding proteins were isolated by affinity chromatography using a derivative of CV (one of the insecticidal compounds) as ligand [10]. Considering the functions of these proteins and the symptoms caused by these compounds, we speculated that V-ATPase and aminopeptidase N (APN)-3 are the putative target proteins. In the present study, we measured the insecticidal toxicity and the enzyme-inhibiting activity of the 12 dihydroagarofuran sesquiterpene polyesters against *M. separata* larvae. The target protein was then validated based on correlation analysis. Results showed that the subunit H of V-ATPase is the target protein of the dihydroagarofuran sesquiterpene polyesters. As with Tulipaline A, as one compound of lactones and aromatic aldehydes that could be exploited as novel nematicdes through inhibiting the activity of V-ATPase [11], this finding also provides ideas for the development of novel pesticides.

## 2. Results

### 2.1. Insecticidal Activity

For the subsequent correlation analysis, the insecticidal activity of 12 dihydroagarofuran sesquiterpene polyesters against *M. separata* larvae was evaluated. Results showed that CV-6-*N*-methylisatoic, CV-6-isobutyric acid ester, CV-6-ketone, NW62, and NW57 did not have insecticidal activity at a dose of 668.45 μg/g, whereas CV, CV-6-α-aminopropanoic acid ester, CV-6-aminoacetic acid ester, wilforine, NW69, NW03, and NW70 were all toxic to the fifth instar larvae. The LD_50_ of the insecticidal compounds was then measured (Table 1). Among the seven insecticidal compounds, the LD_50_ value of wilforine was the lowest. CV-6-α-aminopropanoic acid ester and NW70 also had high toxicity with LD_50_ of 33.605 and 86.271 μg/g, respectively, whereas CV and CV-6-aminoacetic acid ester had relatively lower insecticidal activity.

### 2.2. Effects on the Activity of APN and V-ATPase Activity

#### 2.2.1. Effects on the Activity of APN

**Figure 1:**
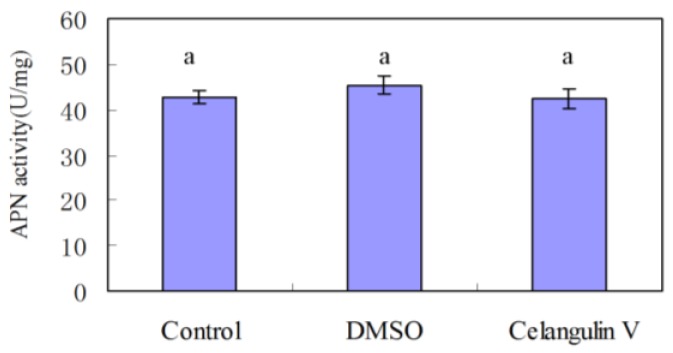
Activity of aminopeptidase N (APN). Results are shown as mean ± SE. The bars show the ranges of standard errors. Letters on the error bars indicate the significant differences by ANOVA analysis (*p* < 0.05).

Our previous study indicated that the symptoms caused by dihydroagarofuran sesquiterpene polyesters are similar to those of Bt toxin; APN is the receptor of Bt toxin [12,13,14]. Here, we chose CV as the representative compound to measure the effect on APN activity. The results showed that APN activity of the group treated with CV has no significant difference with that of the group treated with DMSO. Thus, CV had no effect on the activity of APN (Figure 1).

#### 2.2.2. Effects on the Activity of V-ATPase

The results of the effects of the 12 dihydroagarofuran sesquiterpene polyesters on the V-ATPase activity of *M. separata* larvae are shown in Table 2.

Table 2 shows that the positive control, bafilomycin A1, has an inhibition rate of 48.29% at a dose of 3 μM. Among the dihydroagarofuran sesquiterpene polyesters, wilforine displays high inhibitory effect against V-ATPase, with an inhibition rate of 54.78% at a concentration of 100 μM. CV, CV-6-aminoacetic acid ester, CV-6-α-aminopropanoic acid ester, NW03, NW69, NW70, CV-6-*N*-methylisatoic, and CV-6-isobutyric acid ester showed relatively lower inhibition rate. Compounds NW57, NW62, and CV-6-ketone barely had any effect on V-ATPase.

**Figure 2:**
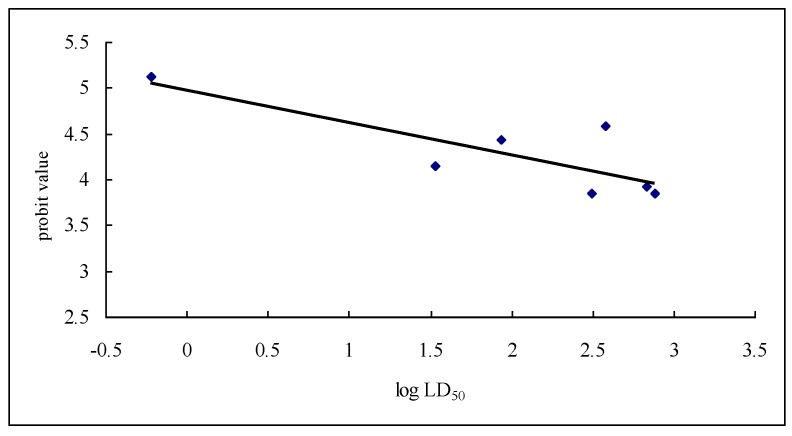
Correlation curve of LD_50_ and inhibition rate of V-ATPase.

Comparison of Table 1 and Table 2 shows that, in general, the tested dihydroagarofuran sesquiterpene polyesters that had high insecticidal activity also had high inhibitory effect on V-ATPase. Moreover, correlation analysis demonstrated that the Pearson correlation coefficient between the LD_50_ and the probit value of the inhibition rate was −0.816 ,which was significant at 0.05 significance level (two-tailed) (*p* = 0.025; Figure 2).

### 2.3. Interaction between Subunit H and Dihydroagarofuran Sesquiterpene Polyesters

From the results of V-ATPase assay and the correlation analysis, we could roughly conclude that V-ATPase was the target protein of dihydroagarofuran sesquiterpene polyesters. However, the proteins separated by affinity chromatography include subunit a, B, and H of V-ATPase. Subunit H of V-ATPase is essential for the catalysis but not for the assembly of the enzyme [15,16,17]. Furthermore, it acts as an inhibitor of ATP hydrolysis in the free V_1_ complex [16], and is probably the binding site for other proteins which interact with V-ATPase [17,18]. Owing to the important function of subunit H, we firstly cloned, expressed, and purified subunit H to study the interaction between subunit H and the 12 dihydroagarofuran sesquiterpene polyesters.

#### 2.3.1. Expression and Purification of Subunit H of V-ATPase

**Figure 3:**
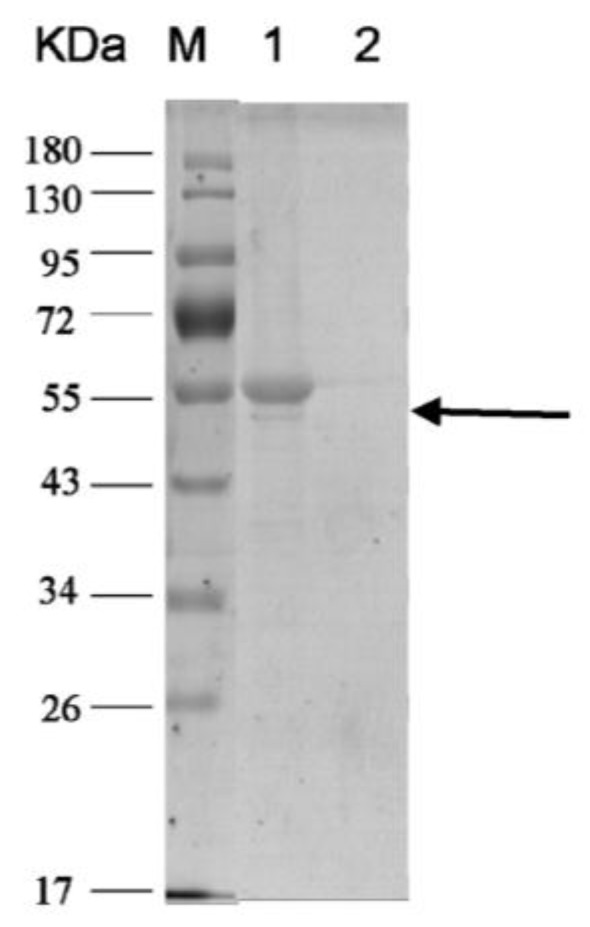
Recombinant subunit H purified by Ni-NTA. M: Protein Marker; 1: concentrated subunit H purified by Ni-NTA; 2: protein flow through the ultramembrane.

Subunit H was expressed at 18 °C after inducing by isopropyl-β-d-thiogalactoside (IPTG) for 18 h. After twice purification by using Ni-NTA, subunit H (~55 kDa) was obtained and concentrated using an ultra membrane (Figure 3). Figure 3 shows that the purity of recombinant subunit H can meet the requirements for interaction analysis.

#### 2.3.2. Interaction between Subunit H and Small Molecules

After purifying the subunit H, the interaction between subunit H and dihydroagarofuran sesquiterpene polyesters was evaluated by microscale thermophoresis (MST, Monolith NT.115). The results indicated that four compounds cannot bind with the recombinant subunit H, whereas the others can bind with subunit H; *K*_D_ values were obtained through the binding curve (Table 3).

**Figure 4:**
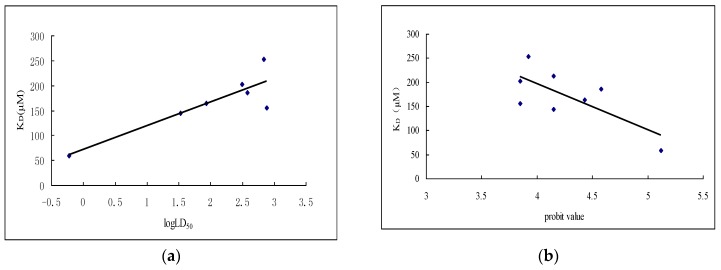
(**a**) Correlation curve of *K*_D_ and LD_50_; (**b**) Correlation curve of *K*_D_ and probit value of inhibition rate of V-ATPase.

Based on the data in Table 1, Table 2 and Table 3, the correlation of *K*_D_ and insecticidal toxicity, as well as the inhibition rate of V-ATPase activity, were analyzed. First, the logarithm value of LD_50_ and *K*_D_ can be fitted in the regression curve *y* = 53.174*x* + 47.634. The Pearson correlation coefficient was 0.870, and the *p*-value was 0.011 (two-tailed), which is significant at 0.05 significance level (Figure 4a). Second, the corresponding probit values of inhibition rate of V-ATPase and *K*_D_ value also were also correlated by fitting in the regression equation of *y* = −95.912*x* + 580.47. The Pearson correlation coefficient was −0.730, which is significant at a 0.05 significance level (*p* = 0.04) (Figure 4b).

CV-6-*N*-methylisatoic and CV-6-isobutyric acid ester were exceptions among all these compounds. CV-6-*N*-methylisatoic did not show insecticidal activity, but it can inhibit the activity of V-ATPase, with an inhibition rate of 24.04% and bind with subunit H with a *K*_D_ value of 213 μM. Whereas, CV-6-isobutyric acid ester did not show toxicity to *M. separata* larvae and cannot bind with subunit H, but it showed low inhibitory activity of V-ATPase. The possible reason is that the insecticidal activity was conducted *in vivo*, where penetration, storage, and degradation by detoxification enzymes occur, thereby inhibiting the insecticidal activity. By contrast, the experiments of V-ATPase activity and interaction with subunit H were conducted *in vitro*, where the compounds form direct contact with proteins.

Except for wilforine, the other dihydroagarofuran sesquiterpene polyesters had lower inhibition rate against V-ATPase from midgut of *M. separata* larvae. One of the possible reasons is that the insecticidal compounds are polyesters, which have poor solubility in aqueous solution. Owing to the difficulty of increasing the solubility, these compounds showed lower inhibition of V-ATPase. In addition, the low solubility of dihydroagarofuran sesquiterpene polyesters may lead to inaccuracy of measurement of *K*_D_.

## 3. Discussion

Correlation analysis is one of the methods used for target validation. The goal of correlation analysis is to analyze the correlation between the different affinities for target proteins after getting a series analog of small molecules of interest and the potency of causing the corresponding phenotype by these molecules. The keys in applying this method is to synthesize analogs of the bioactive small molecule, which has a wide range of activity (IC_50_ or LD_50_ values) spanning at least three orders of magnitude and to make the values more or less equally distributed over those three orders of magnitude of activity instead of being concentrated around one particular value [19]. In general, synthetic bioactive molecules can easily meet the above requirements; by contrast, obtaining sufficient numbers of natural bioactive products for analysis is difficult. In this study, the insecticidal activity (LD_50_ value) of the dihydroagarofuran sesquiterpene polyesters vary in three orders of magnitude, but the number is insufficient.

One important function of midgut epithelial cells of Lepidoptera insect is to transport K^+^ from the haemolymph to the gut lumen. The process is performed by V-ATPase, which is located at the goblet cell apical membranes, as well as the V-ATPase’s partner, the K^+^/*n*H^+^ antiporter [20]. Then, the combined action of the V-ATPase and K^+^/2H^+^ antiporter generates a transepithelial voltage [21]. Herein, the measurement of transmembrane potential difference can indicate the effect of a bioactive molecule on V-ATPase. We evaluated the influence of CV on transmembrane potential of goblet cell apical membranes of the sixth instar larvae of *M. separata* Walker. The results demonstrated that CV can induce depolarization of midgut apical membranes potential, that is, a decrease of the potential difference, which is another indication that CV can inhibit V-ATPase activity [22].

In addition to correlation analysis, genetic approaches can be employed for target validation. Through binding to a specific protein, the bioactive small molecule causes a cellular phenotype and loss of its function. Thus, over-expression of the target protein produce resistance to the small molecule; deletion of the target protein causes the same cellular phenotype with the small molecule; and reduction of the target protein causes hypersensitivity to the small molecule [19]. Subunit H has physiological function only after assembling with other subunits because V-ATPase is a multi-subunits enzyme. The over-expression of subunit H cannot induce the excess assembly of V-ATPase, which then causes resistance to the small molecule, whereas the reduction of subunit H decreases the assembly of V-ATPase, which causes hypersensitivity to small molecules. By using RNAi technology, we injected the dsRNA of subunit H into the third instar larvae of *M. separata* Walker, which significantly decreased the expression of subunit H. Moreover, silencing of the genes of subunit H induced the death of larvae, which showed the same symptoms as those induced by CV and other dihydroagarofuran sesquiterpene polyesters [23].

The target validation showed that subunit H of V-ATPase is one of the target proteins of the insecticidal dihydroagarofuran sesquiterpene polyesters. Based on the above results, we speculate that the mechanism of action of the insecticidal dihydroagarofuran sesquiterpene polyesters. Small molecular compounds are taken by larvae and then transported from the peritrophic membrane to the midgut cells. Then, the small molecules bind with subunit H of V-ATPase on the plasma membrane, which impacts the assembly of subunit H and other subunits so as to inhibit the activity; or the subunit H with small bioactive molecules become a part of V-ATPase, which lead to the malfunction of the whole enzyme. According to the function of V-ATPase, inhibition of its activity may lead to three kinds of results: (1) Amino acid cannot be transported to the gut cells, thereby influencing the protein synthesis and the function of the whole cell; (2) The high alkaline environment in the midgut cannot be maintained, thereby hindering numerous enzymes that function in high-pH environment; and (3) K^+^ cannot enter the gut lumen from cells, thereby inducing accumulation of K^+^ in cells. As a result, the osmotic pressure of cells will be imbalanced and excessive amount of water will enter the cells, resulting in swollen and cracked cells. Subsequently, the haemolymph will enter the gut lumen, which might be the reason for the loss of body fluid.

## 4. Conclusions

The correlation analysis of target protein in the current study, along with the RNAi and electrophysiologic assays in a previous study, showed that subunit H of V-ATPase is the target protein of insecticidal dihydroagarofuran sesquiterpene polyesters.

## 5. Materials and Methods

### 5.1. Insects

Laboratory-adapted *M. separata* (Walker) was obtained from the Institute of Pesticide Science, Northwest A & F University (NWAFU, Yangling, China). The strain was reared on wheat and corn leaves under laboratory conditions for about 20 years, and was never treated with insecticides.

### 5.2. Chemicals

**Figure 5:**
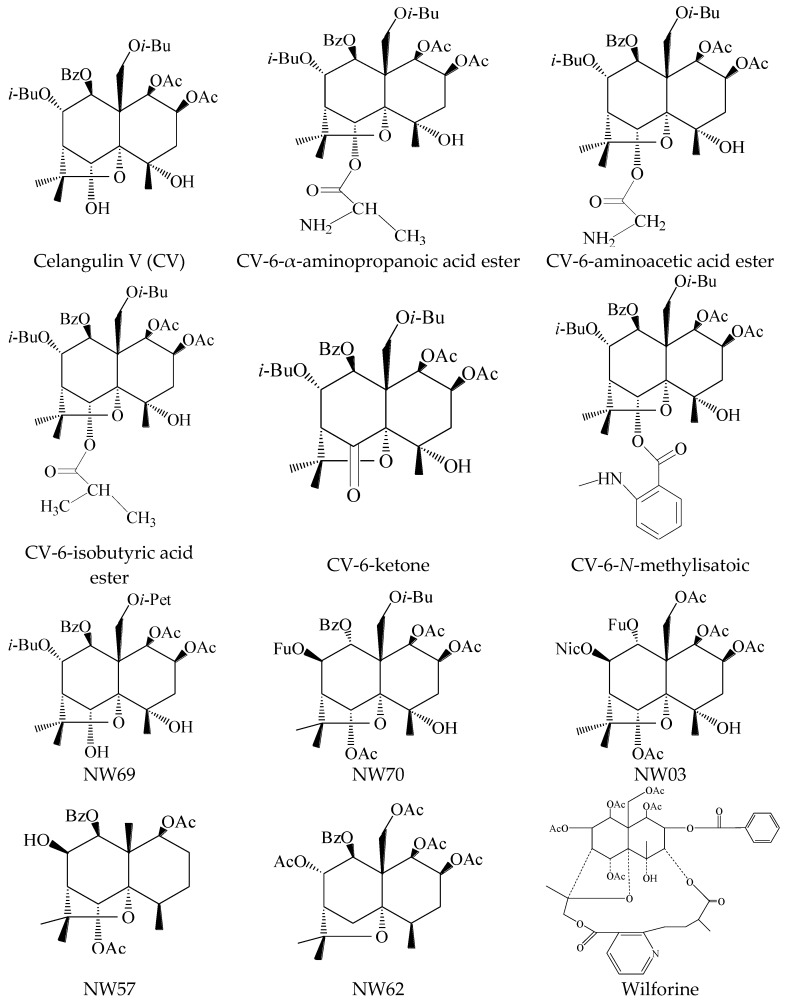

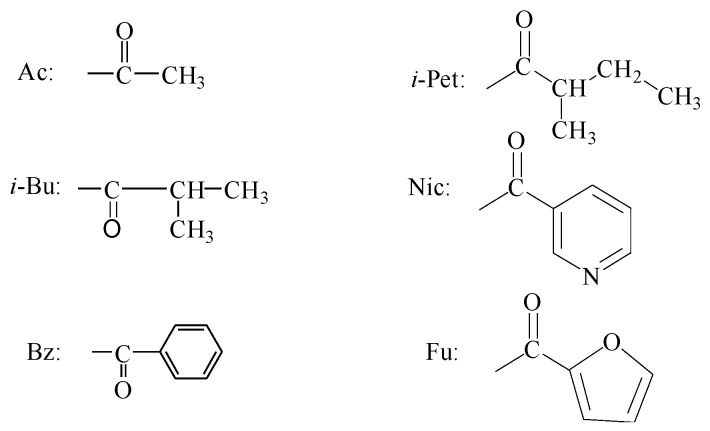
Structures of dihydroagarofuran sesquiterpene polyesters.

CV and its derivatives and analogs (purity >98% according to HPLC analysis) were provided by the Institute of Pesticide Science, NWAFU. Wilforine was purchased from PureOne Biotechnology (Shanghai, China). The structures of all the 12 compounds are presented below (Figure 5). Tris-ATP, orthovanadate, HEPES, NaHCO_3_, KCl, CaCl_2_, and Mes were purchased from Sigma (Shanghai, China). Protease inhibitor cocktail (EDTA-free) was purchased from Roche (Shanghai, China). Bafilomycin A1 was purchased from Santa Cruz Biotechnology (Shanghai, China). Ether was purchased from Xilong Chemical (Shantou, China). The substrate of APN, L-leucine-*p*-nitroanilide (Leu-pNA), was purchased from J & K Chemical Ltd. (Shanghai, China). Tris, NaCl, HCl, EDTA, MgSO_4_, and all other chemicals were purchased from Guanghua Sci-Tech (Guangzhou, China).

### 5.3. Bioassay of Insecticidal Activity

Force-feeding bioassay was conducted to test the insecticidal activity of the 12 dihydroagarofuran sesquiterpene polyesters against the fifth instar larvae of *M. separata* [24]. The 12 tested compounds were dissolved in DMSO and diluted to 5 concentrations by using serial dilution method. Second-day fifth instar larvae were selected and narcotized by cotton ball dipped with ether in Petri dish. Then, 0.5 μL of tested compounds were pipetted into the mouthparts of each larva. The larvae were then transferred into a 24-well plate after swallowing the compounds. For each concentration of each compound, 12 larvae were tested with three replicates. The symptoms presented by the larvae were observed after forced feeding, and the mortality was calculated 24, 48, and 72 h later. At the same time, 20 larvae were randomly selected and weighed to calculate the average body weight. Finally, the LD_50_ (μg/g) of each compound was calculated according to the LC_50_ value, volume of compounds, and average weight of larvae.

### 5.4. APN and V-ATPase Activity Assays

#### 5.4.1. APN Activity Assays

The midgut BBMV of *M. separata* was isolated according to the MgCl_2_ precipitation method [25], as modified by Ferre *et al.* [26]. The final pellet was dissolved in buffer C (150 mM NaCl, 5 mM EGTA, 1 mM PMSF, 20 mM Tris-HCl, and 1% CHAPS) [27]. The protein concentration of BBMV was measured by Bradford Assay.

APN activity was measured according to the method published by Liang *et al.* [28], Watanabe *et al.* [29], Takesue *et al.* [30], and Silva-Filha *et al.* [31]. The enzyme assay system includes 1 mL of buffer (0.25 mol/L Tris-HCl, 26 M NaCl, pH 7.8), 10 μL of BBMV extract, and 1.6 μL of CV (30.2 mM, dissolved in DMSO). The final concentration of CV in the reaction solution is 47 μM according to the amount added into the buffer, as some CV has dissolved out from the buffer. After incubation of the mixture for 30 min at 37 °C, 16 μL of substrate (15.96 mg of Leu-pNA was dissolved in 1 mL of methanol) was added. The absorbance at 405 nm was measured after incubation for 60 min at 37 °C. The assay was repeated for three times, and DMSO was used as control.

#### 5.4.2. V-ATPase Activity Assays

The V-ATPase activity was measured according to the method published by Tiburcy *et al.* [32]. The tested dihydroagarofuran sesquiterpene polyesters were dissolved in DMSO, and the final concentration of the polyesters in the reaction system was 100 μM. Bafilomycin A1 was used as the positive control with the final concentration of 3 μM.

Midguts of sixth instar larvae of *M. separata* were removed from the larvae in the Ringer solution; the peritrophic membrane, gut contents, and malphigian tubules were discarded. The rear parts of the midguts were placed in ice-cold low-adhesion Eppendorf tubes and frozen in liquid nitrogen. Then, the midguts were homogenized and centrifuged twice according to the method published by Tiburcy *et al.* [32]. The V-ATPase assays were performed with three replications. A 160 μL reaction solution consisted of 50 μL of PO buffer (160 mM Tris-Mes, pH 6.9), 20 μL of MV buffer (30 mM MgCl_2_ and 0.8 mM sodium orthovanadate), 20 μL of KN buffer (160 mM KCl and 4 mM NaN_3_), and 10 μL of DMSO/Bafilomycin A1/tested compounds. After 10 min of pre-incubation at 30 °C, 20 μL of Tris-ATP (8 mM) was added to start the reaction. The reaction was stopped after 60 min of incubation at 30 °C by freezing the samples in liquid nitrogen. To measure the V-ATPase activity, the produced inorganic phosphate was determined as described by Wieczorek *et al.* [33].

### 5.5. Binding Affinity Measurements

#### 5.5.1. Expression and Purification of Subunit H of V-ATPase

The gene of V-ATPase subunit H of *M. separata* was cloned through RACE technology [34]. To obtain enough recombinant protein for the binding affinity measurements, the CDS of V-ATPase subunit H was cloned to the pET-15b vector by adding a His tag at the *N*-terminal and then heterologously expressed in *E. coli* BL21 (DE3). The construction of expression vector was described by Li *et al.* [35]. When the OD_600_ value of media reaches ~0.6, IPTG was added into the LB broth at a final concentration of 0.4 mmol/L to induce the expression of recombinant protein at 18 °C for 18 h.

Afterward, the bacterial cells were collected by centrifugation and resuspended with lysis buffer (20 mmol/L Tris-HCl, 300 mmol/L NaCl, and 5 mmol/L imidazole). Lysozyme and nuclease were added into the solution, followed by ice incubation for 30 min. Then, the cells were lysed by ultrasonic. After centrifugation at 12,000 rpm for 30 min (4 °C), the supernatant was used to purify the recombinant protein by Ni-NTA.

The supernatant was loaded onto the Ni-NTA column, which was then washed using buffer (20 mmol/L Tris-HCl, 300 mmol/L NaCl, pH 8.0), with 20 mmol/L and 40 mmol/L imidazole in sequence. An elution buffer (20 mmol/L Tris-HCl, 300 mmol/L NaCl, 500 mmol/L imidazole, pH 8.0) was then used to elute all the binding proteins. SDS-PAGE electrophoresis analysis showed that the eluate proteins washed by the elution buffer contained a number of unspecific proteins. Therefore, a second Ni-NTA purification was necessary. After dialysis at 4 °C for 24 h, the eluate was loaded to the Ni-NTA column. Then, each fraction washed by buffer (20 mmol/L Tris-HCl, 300 mmol/L NaCl, pH 8.0) containing 40, 80, 100, and 200 mM imidazole were collected and detected by SDS-PAGE electrophoresis (Liuyi Biotechnology, Beijing, China).

#### 5.5.2. Interaction between Subunit H and Small Molecules

The purified recombinant protein was concentrated using an ultra membrane (Merck Millipore Amicon, Shanghai, China), and then diluted to 16 μM. After that, the protein was labeled by RED-NHS, according to the user′s manual of Monolith NT.115 (Nano Temper, Munich, Germany). The labeled protein sample was diluted by buffer (20 mmol/L Tris-HCl, 300 mmol/L NaCl, pH 8.0) to ensure the florescence value was between 200 and 1500. Furthermore, Tween-20 (0.1% final concentration) was used to enhance the sample quality. Each dihydroagarofuran sesquiterpene polyester was resolved in DMSO, and 16 different concentrations were prepared by serial dilution. After mixing the 16 samples with labeled protein at a ratio of 1:1, MST capillaries were filled and placed on a tray for the binding affinity measurements. The *K*_D_ values of each compound with the subunit H were calculated according to the binding curve [36].

## Figures and Tables

**Table 1:** Insecticidal activity of dihydroagarofuran sesquiterpene polyesters against the fifth instar larvae of *M. separate*.

Samples	Regression Equation	Correlation Coefficient	LD_50_ (μg/g)	95% Confidence Limits (μg/g)
Celangulin V (CV)	*Y* = 3.6544 + 0.47509*x*	0.9813	679.479	171.7784–2687.7183
CV-6-α-aminopropanoic acid ester	*Y* = 1.9537 + 1.99575*x*	0.9488	33.605	27.1526–41.5919
NW03	*Y* = 2.9385 + 0.79935*x*	0.9922	379.286	173.1693–830.7353
NW70	*Y* = 1.3655 + 1.87692*x*	0.9829	86.271	60.8162–122.3788
NW69	*Y* = −3.6165 + 3.45942*x*	0.9912	309.559	257.2790–372.4620
Wilforine	*Y* = 5.3414 + 1.52479*x*	0.9972	0.597	0.3752–0.9506
CV-6-aminoacetic acid ester	*Y* = 3.2465 + 0.6077*x*	0.9805	767.811	189.6674–894.5342

**Table 2:** Effects of dihydroagarofuran sesquiterpene polyesters on V-ATPase from the midgut of *M. separata* larvae.

Compounds	Concentration (μM)	Specific Activity (nmol Pi/min/mg)	Inhibition Rate (%)
DMSO	-	451.65 ± 19.2740 ^a^	-
Bafilomycin A1	3	233.52 ± 14.3258 ^h^	48.29
Celangulin V (CV)	100	387.57 ± 12.6793 ^cde^	14.19
CV-6-aminoacetic acid ester	100	394.19 ± 29.1940 ^bcd^	12.72
CV-6-α-aminopropanoic acid ester	100	362.05 ± 21.6747 ^bcd^	19.83
Wilforine	100	204.25 ± 34.6747 ^h^	54.78
NW03	100	298.19 ± 12.3455 ^g^	33.98
NW69	100	394.61 ± 35.3014 ^bcd^	12.63
NW70	100	317.80 ± 17.9918 ^fg^	29.64
NW57	100	443.07 ± 34.4936 ^abc^	1.90
NW62	100	420.49 ± 5.5092 ^ab^	6.90
CV-6-*N*-methylisatoic	100	343.06 ± 11.3159 ^efg^	24.04
CV-6-isobutyric acid ester	100	396.10 ± 11.2234 ^cd^	12.30
CV-6-ketone	100	421.308 ± 27.3712 ^abc^	6.72

Note: different lowercase letters (a, b, c, d, e, f, g, h) indicate the significant differences by ANOVA analysis (*p* < 0.05).

**Table 3:** Nteraction between dihydroagarofuran sesquiterpene polyesters and subunit H of *M. separata*.

Samples	Interaction	*K*_D_ Value (μM)
Wilforine	binding	59.0
CV-6-α-aminopropanoic acid ester	binding	144.0
CV-6-aminoacetic acid ester	binding	156.0
NW70	binding	164.0
NW69	binding	186.0
NW03	binding	203.0
CV-6-*N*-methylisatoic	binding	213.0
Celangulin V (CV)	binding	253.0
CV-6-isobutyric acid ester	no-binding	-
CV-6-ketone	no-binding	-
NW57	no-binding	-
NW62	no-binding	-

## Abbreviations

The following abbreviations are used in this manuscript:

RACE: Rapid-amplification of cDNA ends
CDS: Coding DNA Sequence
DMSO: dimethylsulfoxide
anova: Analysis of Variance
